# Productive Performance, Egg Quality and Yolk Lipid Oxidation in Laying Hens Fed Diets including Grape Pomace or Grape Extract

**DOI:** 10.3390/ani12091076

**Published:** 2022-04-21

**Authors:** Carlos Romero, Ignacio Arija, Agustin Viveros, Susana Chamorro

**Affiliations:** 1 Faculty of Sciences and Arts, Universidad Católica Santa Teresa de Jesús de Ávila (UCAV), Calle Canteros s/n, 05005 Ávila, Spain; carlos.romero@ucavila.es; 2 Department of Animal Production, Faculty of Veterinary Medicine, Universidad Complutense de Madrid, 28040 Madrid, Spain; arijai@vet.ucm.es (I.A.); viverosa@vet.ucm.es (A.V.); 3 Physiology and Microbiology (Animal Physiology Unit), Department of Genetics, Faculty of Biology, Universidad Complutense de Madrid, 28040 Madrid, Spain

**Keywords:** grape polyphenols, grape pomace, grape extract, egg quality, antioxidant activity, laying hens

## Abstract

**Simple Summary:**

Phenolic compounds from grape products are known to possess antioxidant activity. Furthermore, grape polyphenols (anthocyanins) could be valorised as natural pigments. However, the dietary use of grape products in laying hen husbandry and its effect on egg quality traits and yolk lipid oxidation have been scarcely studied. In the present research work, egg yolk colour and Haugh units were increased with the intake of grape pomace and extract. Moreover, dietary inclusion of grape pomace at 60 g/kg increased the proportion of polyunsaturated fatty acids in the yolk and improved the yolk lipid oxidative stability during the storage of eggs, whereas no effect was obtained with grape extract. Regarding hen performance, both grape products reduced feed intake, feed conversion ratio and average egg weight, but they did not affect daily egg production and mass. Feeding grape pomace, at 60 g/kg, and grape extract, either at 0.5 or 1.0 g/kg, decreased protein digestibility. In conclusion, the addition of grape products in the diet of laying hens improved the egg quality but reduced feed intake and egg weight. Dietary grape pomace showed higher antioxidant potential in egg yolk than grape extract.

**Abstract:**

An experiment was conducted to assess in laying hens the effect of including grape pomace (GP, at 30 or 60 g/kg) or grape extract (GE, at 0.5 or 1.0 g/kg) on egg production, feed conversion ratio, protein and polyphenol digestibility, egg weight, egg quality, yolk fatty acid profile and oxidative stability of yolk lipids. No differences were detected among diets for egg production (83.8%, on average) or egg mass (56.8 g/d, on average). However, the average egg weight was lower (*p* = 0.004) for dietary treatments GP 30, GP 60 and GE 0.5 (67.5 g, on average) than for control hens (68.5 g). Accordingly, in hens fed the GP diets the proportion of XL eggs was lower (*p* = 0.008) than in control hens, while the proportion of M eggs was higher (*p* < 0.001) in hens fed the diets GP 30, GP 60 and GE 0.5 than in the control group. The dietary inclusion of both GP and GE decreased daily feed intake (120.9 vs. 125.3 g/d, *p* < 0.001) and the feed conversion ratio (2.09 vs. 2.18, *p* = 0.01). Feeding GP at 60 g/kg or GE reduced excreta protein digestibility (54.7 vs. 62.8%, *p* < 0.001), whereas all GP and GE diets showed higher excreta polyphenol digestibility than the control treatment (57.2 vs. 41.0%, *p* < 0.001). While yolk colour score was increased with all grape diets (8.12 vs. 7.34, *p* < 0.001), the dietary inclusion of GP, either at 30 or 60 g/kg, and that of GE at 1.0 g/kg increased the Haugh units of the albumen (80.8 vs. 76.4 Haugh units, *p* = 0.001). Shell thickness remained unaffected by dietary treatments (365.2 μm, on average). When included in the diet at 60 g/kg, GP reduced the proportion of saturated fatty acids in the yolk (31.6 vs. 32.9%, *p* = 0.001) and that of monounsaturated fatty acids (39.5 vs. 41.4%, *p* < 0.001), while it increased the percentage of polyunsaturated fatty acids (28.9 vs. 25.7%, *p* < 0.001). In fresh eggs, no significant differences were found for the malondialdehyde (MDA) concentration (0.146 mg/kg, on average). In stored eggs, the MDA amount was lower in the eggs of the laying hens fed GP at 60 g/kg than in the eggs of the control hens (1.14 vs. 1.64 mg/kg, *p* = 0.025). In conclusion, the inclusion of grape pomace, either at 30 or 60 g/kg, and grape extract at 1.0 g/kg in the diet of laying hens improved some egg quality traits, but feeding grape pomace resulted in a lower average weight of eggs. Nevertheless, feeding laying hens with diets containing grape pomace resulted in a higher antioxidant potential in egg yolk than dietary inclusion of grape extract.

## 1. Introduction

Hen eggs present a high nutritional value resulting from their varied composition: high concentration of essential amino acids, low calorie content, richness in vitamins A, E, B_7_ and B_12_ and high content of zinc, iron and phosphorus [1,2,3]. Furthermore, eggs can be deemed as functional food because of their content of bioactive compounds with nutraceutical properties such as phospholipids, ovalbumin, ovotransferrin, selenium and choline [4,5]. As a result, moderate egg consumption has been proven to exert health benefits in human beings including prevention of hypertension, anti-ageing effect and improvement of ocular health [6,7,8]. Human consumption of hen eggs is steadily increasing worldwide, with an increase of 14.1% of global per capita egg consumption from 2010 to 2019 [9].

Still, hen’s egg nutritional value can be further enhanced by increasing egg concentration in polyunsaturated fatty acids (PUFA), whose intake has been associated with a lower incidence of cardiovascular diseases [10] and the improvement of cognitive abilities [11]. Enrichment of hen eggs with PUFA can be achieved by feeding laying hens with diets including sources of PUFA, such as linseed oil or cod liver oil [12,13]. Nevertheless, unsaturated fatty acids are highly susceptible to lipid oxidation, which makes PUFA-enriched eggs more perishable and shortens their shelf life [14,15,16]. Moreover, oxidation of yolk lipids can depreciate eggs because it causes bad taste and unpleasant odour [17].

Peroxidation of egg lipids has traditionally been mitigated with the inclusion of antioxidant supplements such as alpha-tocopheryl acetate in the diet of laying hens [18]. However, alternative compounds with antioxidant properties such as polyphenols could also be included in hens’ diets in order to improve stability of egg lipids [19]. Indeed, phenolic compounds originating from grape (mainly, catechins and proanthocyanidins) have shown to possess the capacity to scavenge free radicals and terminate oxidative reactions [20]. In this sense, several studies [21,22,23,24] have reported that the dietary inclusion of grape by-products (grape pomace, grape seeds and grape skins) has been effective in reducing lipid oxidation in the meat of broiler chickens, but there is a dearth of works evaluating the effect of dietary inclusion of grape products on yolk lipid oxidation [25].

Furthermore, synthetic pigments used to increase egg yolk colour score could be partly spared as additives in laying hens’ diets by means of dietary incorporation of grape products, since grape anthocyanins are the natural pigments responsible for the red-purple colour in grapes [26,27]. Despite this, very little information is currently available about the inclusion of grape products in the diet of laying hens and its effect on the quality traits of eggs [28]. The few research works existing on this topic have considered dietary doses of grape pomace ranging from 20 to 60 g/kg [25,29] and doses of grape seeds from 5 to 30 g/kg [30,31]. Previous studies in chickens have highlighted the importance of the grape source [23,24], since it has been demonstrated that the antioxidant potential of grape polyphenols and their effect on productive performance depend not only on their dietary dose but also on the polyphenol source. Depending on the grape products included in the diet, the same dietary dose of grape polyphenols may be detrimental for birds’ productive performance or not. To our knowledge, there is no published study jointly comparing the antioxidant potential and the effect on egg production of different sources of grape polyphenols in laying hens.

Thus, the aim of the present work was to evaluate the effect of the dietary inclusion of grape pomace (at 30 and 60 g/kg) and grape extract (at 0.5 and 1.0 g/kg) on egg production, feed conversion ratio, protein and polyphenol digestibility, egg weight, egg quality, fatty acid profile in the yolk and oxidative stability of yolk lipids in laying hens.

## 2. Materials and Methods

### 2.1. Grape Products Tested

Grape pomace (GP) originating from red grapes (*Vitis vinifera* var. Cencibel) was obtained at Explotaciones Hermanos Delgado organic winery (Socuéllamos, Ciudad Real, Spain). Whole GP was dried immediately after pressing in a rotary trammel type dryer with indirectly heated air at temperatures below 80 °C. Afterwards, GP was ground to pass a 1 mm mesh screen and was directly incorporated to the experimental diets. The proximate composition of GP is provided in Table 1.

The water-soluble grape extract (GE) was purchased from Nor-Feed Sud (Angers, France). It contained 30.0 ± 0.93 g gallic acid equivalents/100 g DM of total extractable polyphenols.

### 2.2. Birds and Diets

The present study consisted of a four-week experiment designed with five dietary treatments. Before the beginning of the trial, laying hens were given a two-week adaptation period. Fifty-week-old 375 Hy-Line W36 laying hens (75 hens per treatment) were used. Hens were allocated randomly to 15 wire cages (3 replicate cages per diet; 25 hens per cage) in a completely enclosed fan-ventilated and environmentally controlled building with a daily light programme of 16 h (photoperiod from 06:00 am to 10:00 pm). Cages used in the experiment were enriched cages fulfilling the requirements set by the Council Directive 1999/74/EC (750 cm^2^ of cage area per hen, more than 0.20 m of height at any point of the cage, at least 0.15 m of perch per hen, a nest within the cage, etc.). Experimental procedures were approved by the University Complutense of Madrid Animal Care and Ethics Committee (protocol code CEA-UCM 20/2012) in compliance with the guidelines for the Care and Use of Animals for Scientific Purposes of the Ministry of Agriculture, Fishery and Food.

Ingredient and nutrient compositions of the five experimental diets are shown in Table 2. Diets were formulated to be isonitrogenous and isocaloric and to contain the same amount of calcium, phosphorus and sodium. Celite (Celite Corp., Lompoc, CA, USA), a source of acid-insoluble ash (AIA), was added at 10 g/kg to all diets as an indigestible marker. Feed, which was offered daily in a mash form, and water were provided ad libitum throughout the whole experiment. Experimental diets were as follows: (1) control diet (including 100 mg/kg of vitamin E); (2) diet including 30 g/kg of GP; (3) diet including 60 g/kg of GP; (4) diet including 0.5 g/kg of GE; (5) diet including 1.0 g/kg of GE. The vitamin E (α-tocopheryl acetate) contained in the control diet was provided by DSM Nutritional Products Iberia S.A. (Alcalá de Henares, Madrid, Spain).

### 2.3. Hen Performance Measurement and Egg Quality Assessment

Feed intake by laying hens was measured weekly but then divided by the number of hens per cage and by seven to determine the daily feed intake per bird. Each day, all laid eggs were collected manually, and the number of eggs was recorded per cage. Unmarketable eggs (shell-less eggs, broken eggs, eggs whose shell showed deformities, eggs with abnormal shape or colour) were put aside and counted to determine their proportion over the total number of eggs laid. Eggs suitable for sale were all weighed and graded by weight in keeping with the egg weight classes established by the Commission Regulation No. 589/2008 (XL—very large, L—large, M—medium, S—small). Daily egg mass was calculated per cage by multiplying daily egg production by average egg weight divided by 100. Feed conversion ratio was calculated by dividing feed intake by egg mass.

On the first and second day of week four of the trial, 60 freshly laid eggs were collected daily per treatment (daily, 20 eggs per replicate of each dietary treatment), individually weighed and used for determination of yolk colour score, Haugh units of the albumen and shell thickness. Yolk colour was assessed using the Roche Yolk Colour Fan and data were expressed in the standard DSM Roche Fan values (from 1 for light yellow to 15 for orange). Albumen height of the 120 eggs was measured with a QCH apparatus (TSS, York, UK). Haugh units were calculated thereafter with the formula: Haugh units = 100 × log (*h* − 1.7 × *w*^0.37^ + 7.57), where *h* = albumen height (mm) and *w* = egg weight (g) [32]. Shell thickness was determined at the equator of eggs with a digital Mitutoyo shell thickness micrometre (Kawasaki, Japan).

On the third day of week four of the trial, 12 freshly laid eggs were collected per dietary treatment (4 eggs per replicate of each diet). These eggs were broken and for each one of them the yolk was separated from the egg white. Then, the yolks of two eggs of the same dietary treatment were pooled, hence obtaining six pools of yolks per diet that were frozen at −20 °C, freeze-dried and later on used for the determination of the yolk fatty acid profile.

On the fourth day of week four of the trial, ten freshly laid eggs were randomly collected per dietary treatment. The yolks from two eggs of the same diet were pooled, and in these pools of yolks (five pools of yolks per dietary treatment) the thiobarbituric acid reactive substances (TBARS) were measured on the same day of egg collection (day 0). Additionally, this same day another 18 freshly laid eggs were collected per dietary treatment (6 eggs per replicate of each diet) and were stored at room temperature for four months. After four months of storage, these eggs were broken and the yolks from two eggs of the same diet were pooled. In the nine pools of yolks obtained for each dietary treatment, the TBARS value was determined to assess the extent of yolk lipid oxidation.

### 2.4. Excreta Measurements

On the first day of week three of the trial, two clean stainless steel collection trays were placed contiguously under each cage and excreta from the hens were collected for 48 h. A sample of excreta was taken from each tray (six samples of excreta per dietary treatment), placed in a plastic container and stored at −20 °C. Excreta samples were freeze-dried, ground (1 mm screen) and used to determine celite, protein and total extractable polyphenol (TEP) contents.

### 2.5. Chemical Analyses

Dry matter (930.15), crude protein (976.05), crude fibre (978.10) and gross energy were analysed according to the methods of the AOAC [33]. Neutral-detergent fibre (NDF), acid-detergent fibre (ADF) and acid-detergent lignin (ADL) were determined according to the sequential method of Van Soest et al. [34]. The AIA concentration in diet and excreta contents was measured after ashing the samples and treating the ashes with boiling 4 M HCl [35]. Chemical analyses were performed in triplicate.

#### 2.5.1. Total Extractable Polyphenol Content

For the extraction of polyphenols in GP, GE, diets and excreta, 0.50 g of sample was placed in a capped centrifuge tube, suspended in 20 mL of acidic methanol/water (50:50 *v*/*v*, pH = 2) and thoroughly shaken at room temperature for 1 h. The tube was centrifuged at 3500 rpm for 15 min and the supernatant was separated. Twenty millilitres of acetone/water (70:30 *v*/*v*) was added to the residue and shaking and centrifugation were repeated. The methanol and acetone extracts were combined and used for TEP quantification. The TEP content was determined by Folin–Ciocalteu procedure [36]. Briefly, a mixture of 0.5 mL of extract, 0.5 mL of Folin–Ciocalteu reagent (Sigma-Aldrich, St. Louis, MO, USA) and 10 mL of 1 M Na_2_CO_3_ were introduced in a 25 mL volumetric flask. After reacting for 1 h, absorbance was measured at 750 nm using an ultraviolet-visible spectrophotometer (Hitachi U-2000; Hitachi, Ltd., Tokyo, Japan). Absorbance values were compared against a standard curve made with gallic acid (Sigma-Aldrich, St. Louis, MO, USA) ranging from 0.05 to 0.5 mg of gallic acid per mL. Results were expressed as grams of gallic acid equivalents (GAE) per 100 g of DM.

#### 2.5.2. Fatty Acid Composition

Fatty acids were analysed in duplicate in freeze-dried samples of yolks after submitting the samples to a simplified direct bimethylation procedure. Briefly, 0.1 g of the freeze-dried and ground samples was weighed in duplicate and 2 mL 0.5 M sodium methoxide in anhydrous methanol and 1 mL heptane containing 1 mg/mL C13:0 (≥99.9%) were added, as internal standard. The samples were then heated at 50 °C for 15 min before 2 mL 10% acetyl chloride in anhydrous methanol was added. Next, the samples were mixed thoroughly and heated for 1 h at 60 °C. Heptane (3 mL) and distilled water (1 mL) were added before mixing and centrifuging for 5 min at 1500× *g*. The organic solvent top layer was pipetted into a second tube before further 2 mL of heptane was added to the original tube, mixed and centrifuged as previously. After pooling the organic layers in the second tube, anhydrous sodium sulphate (0.2 g) was added, mixed and then centrifuged. Finally, an aliquot was collected in an amber vial for subsequent gas chromatography analysis. Fatty acid methyl esters (FAME) were performed with a gas chromatograph (Agilent 7820A) equipped with a flame-ionisation detector and an Agilent HP-88 column (60 m × 250 μm × 0.2 μm) with split injection (40:1) and Helium at a constant flow of 1.5 mL/min as the carrier gas. Detector temperature was set at 260 °C and injector oven temperature at 250 °C. The temperature profile of the oven was from 125 °C increasing by 8 °C/min to 145 °C followed by 2 °C/min to 220 °C. Identification was accomplished by comparing the retention times of peaks from samples with those of FAME standard mixtures. Quantification of FAME was based on the internal standard technique and on the conversion to relative peak areas to weight percentage, using the corrected response factor of each fatty acid [37]. Fatty acids were expressed as percentage of the sum of identified fatty acids. For instance, the percentage of saturated fatty acids (SFA) resulted from the sum of the percentages of C14:0, C15:0, C16:0, C17:0, C18:0 and C20:0. The percentage of monounsaturated fatty acids (MUFA) resulted from the sum of the percentages of C14:1, C16:1t, C16:1c, C18:1n9t, C18:1n9c, C18:1n11c and C20:1. The percentage of PUFA resulted from the sum of the percentages of C18:2n6c, C18:3n6, C18:3n3, C20:2, C20:3n6, C20:4n6, C22:5n3 and C22:6n3.

#### 2.5.3. Oxidation Assessment

The extent of yolk lipid oxidation was determined by measuring the TBARS in fresh eggs (day 0) and after four months of storage of eggs at room temperature, using the procedure described by Botsoglou et al. [38] with minor modifications. Two grams of yolk were homogenised with eight millilitres of 5% trichloroacetic acid in an Ultraturrax at 21,280× *g* for 1 min. Butylated hydroxytoluene was added prior to homogenisation at a level of 125 μg/mg of fat. The blended sample was filtered through Whatman number 2V filter (Whatman International Ltd., Maidstone, UK) and 1.5 mL of the filtrate was mixed with 1 mL of 0.8% thiobarbituric acid in distilled water in capped test tubes. Tubes were vortexed, incubated at 80 °C for 30 min and absorbance was determined at 532 nm using an ultraviolet-visible spectrophotometer Hitachi U-2000 (Hitachi, Ltd., Tokyo, Japan). Results were expressed as mg of malondialdehyde (MDA) per kilogram of yolk after the preparation of a standard curve of 1,1,3,3-tetraethoxy propane.

### 2.6. Calculations and Statistical Analysis

Apparent excreta digestibility of crude protein (CP) and TEP was calculated using the following formula:

100% − [100% × (AIA concentration in feed/AIA concentration in excreta) × (CP or TEP concentration in excreta/CP or TEP concentration in feed)]

Data of continuous variables were subjected to a one-way analysis of variance (ANOVA) by using the general linear model procedure (Version 9.4, SAS Institute Inc., Cary, NC, USA). When the effect was declared significant (*p* < 0.05), treatment means were compared using a Duncan’s multiple-range test. The CATMOD procedure of SAS was used to analyse the frequencies of the different size classes of eggs.

The cage with 25 hens constituted the experimental unit for performance parameters (egg production, egg weight, egg mass, feed intake, feed conversion ratio and egg size classes) and for the digestibility assay. The egg was the experimental unit for yolk colour score, albumen Haugh units and shell thickness. A pool of two yolks from eggs of the same dietary treatment represented the experimental unit for analyses of the fatty acid profile and the MDA concentration.

## 3. Results

### 3.1. Hen Performance

As reported in Table 3, no significant differences (*p* > 0.05) were detected among dietary treatments for egg production (83.8%, on average) or egg mass (56.8 g/d, on average). However, the average egg weight was lower (*p* = 0.004) for dietary treatments GP 30, GP 60 and GE 0.5 (67.5 g, on average) than for control hens (68.5 g) or hens fed the GE 1.0 diet (68.6 g). Accordingly, in hens fed the GP diets the proportion of XL eggs was lower (*p* = 0.008) than in control hens, while the proportion of M eggs was higher (*p* < 0.001) in hens fed the diets GP 30, GP 60 and GE 0.5 than in the control group (Table 4). No significant effect of diet (*p* > 0.05) was observed for the proportions of L eggs (71.0%, on average), S eggs (0.220%, on average) or unmarketable eggs (3.21%, on average).

The dietary inclusion of both GP and GE decreased daily feed intake (120.9 vs. 125.3 g/d, *p* < 0.001; Table 3) and feed conversion ratio (2.09 vs. 2.18, *p* = 0.01), as compared with the control group.

### 3.2. Excreta Protein and Polyphenol Digestibility

**Figure 1:**
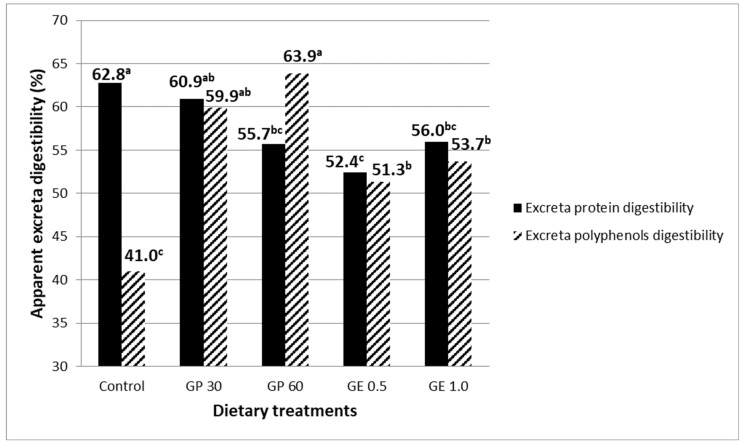
Excreta digestibility of protein (SEM ^1^ = 1.64, ***) and that of total extractable polyphenols (SEM = 2.86, ***) in laying hens fed diets containing grape pomace (GP) or grape extract (GE) at different concentrations. Different letters for the same parameter (a, b, c) indicate significant differences (*p* < 0.05). ^1^ SEM, standard error of means; each value represents the mean of six samples per dietary treatment (two samples per replicate). *** *p* < 0.001.

Feeding GP at 60 g/kg or GE reduced excreta protein digestibility with respect to the control group (54.7 vs. 62.8%, *p* < 0.001; Figure 1). On the contrary, all GP and GE diets showed higher digestibility of excreta polyphenols than the control treatment (57.2 vs. 41.0%, *p* < 0.001).

### 3.3. Egg Quality

All GP and GE diets resulted in higher yolk colour score with respect to the values measured in the eggs of the control hens (8.12 vs. 7.34, *p* < 0.001; Table 5). The dietary inclusion of GP, either at 30 or 60 g/kg, and that of GE at 1.0 g/kg increased the Haugh units of the albumen (80.8 vs. 76.4 Haugh units, *p* = 0.001), as compared with the control treatment. However, no significant effect (*p* > 0.05) was observed among the different dietary treatments for the shell thickness (365.2 μm, on average).

### 3.4. Yolk Fatty Acid Profile

The profile of fatty acids of the yolks is presented in Table 6. The dietary inclusion of GP, either at 30 or at 60 g/kg, reduced the proportion of SFA in the yolk with respect to the control group (31.9 vs. 32.9%, *p* = 0.001). When included in the diet at 60 g/kg, GP did not only lead to a reduction in the proportion of SFA, but it also decreased that of MUFA (39.5 vs. 41.4%, *p* < 0.001) and increased the percentage of PUFA (28.9 vs. 25.7%, *p* < 0.001), as compared with the eggs of the control hens. This increase in the proportion of PUFA in the eggs of hens fed the GP 60 diet was due to an increase in the proportion of ω-6 fatty acids (28.0 vs. 24.8%, *p* < 0.001), since the proportion of ω-3 fatty acids was, on the contrary, reduced (0.617 vs. 0.682%, *p* < 0.001). The dietary inclusion of GE, either at 0.5 or 1.0 g/kg, did not significantly modify the yolk fatty acid profile with respect to the control treatment.

### 3.5. Yolk TBARS Value

**Figure 2:**
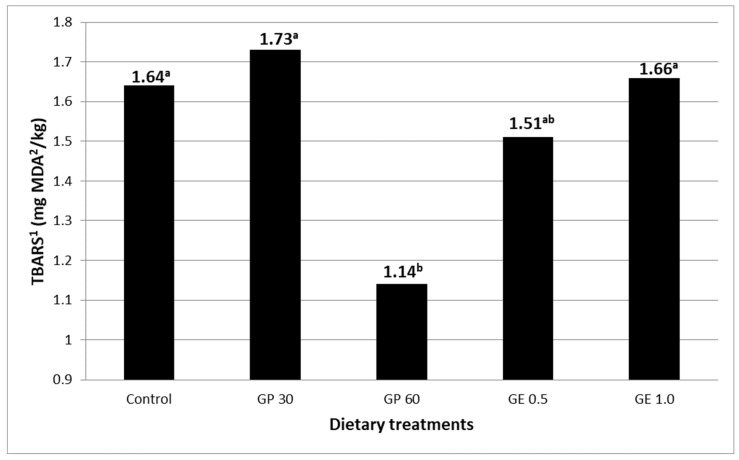
Yolk lipid oxidation (SEM ^3^ = 0.149, *) measured in four-month-stored eggs of laying hens fed diets containing grape pomace (GP) or grape extract (GE) at different concentrations. Different letters (a, b) indicate significant differences (*p* < 0.05). ^1^ TBARS: thiobarbituric acid reactive substances. ^2^ MDA: malondialdehyde. ^3^ SEM, standard error of means; each value represents the mean of nine samples per dietary treatment (three samples per replicate); each sample resulted from the pool of two yolks. * *p* < 0.05.

In fresh eggs (day 0), no significant differences (SEM = 0.010, *p* > 0.05) among the various dietary treatments were found for the MDA concentration (0.146 mg MDA/kg, on average). The storage of eggs increased (*p* < 0.001) the yolk MDA concentration from the average value of 0.146 mg/kg on day 0 to 1.52 mg/kg, on average, in eggs that had been stored for four months at room temperature. The results obtained for the oxidation of yolk lipids after four months of egg storage are reported in Figure 2. In the stored eggs, the MDA amount was lower in the eggs of the laying hens fed GP at 60 g/kg than in the eggs of the control hens (1.14 vs. 1.64 mg MDA/kg, *p* = 0.025). The other grape product treatments did not differ significantly from the control group.

## 4. Discussion

### 4.1. Hen Performance and Protein and Polyphenol Intestinal Utilisation

In the current study, the inclusion of GP or GE in the diets did not affect the egg production or the egg mass of the laying hens. Similarly, Kara et al. [25] and Reis et al. [29] reported that the inclusion of GP from 20 to 60 g/kg in the diet of laying hens did not affect egg production with respect to hens from the control group. Sun et al. [39] also did not find any effect on the laying rate of hens when feeding grape seed extract. Research on the dietary use of grape products in laying hens is rather scarce, but studies testing the dietary effect of grape by-products on the growth performance of broiler chickens are more numerous. In works conducted with broiler chickens, it was observed that chicken productive performance was not affected as long as the dietary inclusion rate of GP was up to 50 g/kg [40,41]. However, with higher dietary doses of grape by-products (for instance, with grape skins added in the diet of chickens at 60 or 110 g/kg, [22,23]), the weight gain of chickens was impaired. Nonetheless, the effect of grape polyphenols on chicken growth performance depends both on their dietary dose and on their source [19]. With diets containing the same amount of grape polyphenols (2.4 g/kg) but differing in the source of these polyphenols (GP, grape seeds or grape skins), chicken weight gain was reduced by the diet including grape skins but was not affected by the other grape by-product diets [23]. Furthermore, when feeding grape extract [42], the negative effect on chicken growth rate was already detected from a grape polyphenol concentration of 1.48 g/kg. The detrimental effects on productive performance observed with diets containing high concentrations of grape polyphenols are usually attributed to the reduction in protein digestibility [28], since among these phenolic compounds there are condensed tannins that bind to proteins and thereby hinder protein digestion. Even if egg production was not impaired by the dietary inclusion of grape products in the present research work, it is, nonetheless, true that excreta protein digestibility of laying hens was decreased when birds were fed GP or GE. This reduction in protein digestibility did not cause a reduction in the number of eggs laid but may have accounted for the lower egg weight detected in several experimental groups (hens fed the GP 30, GP 60 and GE 0.5 diets). Actually, as shown by Shim et al. [43], the weight of eggs laid by commercial layers is strongly influenced by the protein intake. The latter authors showed that there exists a linear direct relationship in laying hens between the egg weight and the dietary protein level. In their study, egg weight decreased proportionally to the reduction in the dietary protein percentage. In addition, in agreement with the findings of our work, Froes et al. [44] detected that egg weight of quails linearly decreased as dietary GP level increased from 20 to 60 g/kg, and Kaya et al. [30] reported lower egg weight in laying hens when diets contained grape seeds at 15 g/kg.

In our work, it was observed that feed intake of laying hens was decreased by the inclusion in the diet of both grape products. Taking into account that the dietary inclusion of grape polyphenol sources had no influence on egg production, the reduction in feed intake resulted in an improvement of feed conversion ratio in hens fed the GP and GE diets. In quails fed diets including grape seeds, Silici et al. [45] also found an enhancement of feed conversion efficiency.

The average value found in the present study for excreta digestibility of TEP in hens fed the grape treatments was 57.2%. This result indicates that laying hens are able to utilise grape phenolic compounds in the intestine. To the authors’ knowledge, this is the first research work in which the digestibility of polyphenols was measured in laying hens; consequently, there are no reference values with which the results of TEP digestibility of the current study could be compared. Nevertheless, it could be pointed out that the results of excreta TEP digestibility found here for the laying hens fed the grape products diets fall within the range of values of excreta TEP digestibility obtained in experiments conducted with broiler chickens that were fed diets containing GP, GE or grape skins [22,23,40,46].

### 4.2. Egg Quality Parameters

Feeding laying hens with diets containing grape polyphenol sources had no effect on shell thickness but it increased the yolk colour score and the Haugh units of the albumen. Likewise, no effect on egg shell thickness was found due to the dietary inclusion of GP in laying hens [25] or in quails [44] or with that of grape seeds in these same species [30,45].

Little information is available about the effect of feeding grape products on yolk colour and Haugh units in laying hens. While Kara et al. [25] found no significant effect of feeding up to 60 g/kg of GP on egg yolk colour in laying hens, Froes et al. [44] found a positive quadratic relationship between yolk pigmentation and GP dietary level in quails. Similarly, Omri et al. [47] concluded that feeding a diet containing tomato paste and red pepper to laying hens increased the Roche colour score and the redness in yolks of fresh eggs, as compared with the control diet. Grapes, as well as tomato and red pepper, are natural sources of β-carotene and lutein [48], which are pigmenting substances commonly used in European feed mills to achieve the optimal yolk colouration desired by customers (usually, reddish orange yolks, i.e., from 10 to 14 in the Roche Yolk Colour Fan; [49]). Thus, the inclusion of grape products in the diet of laying hens could contribute to somewhat reduce the dietary use of synthetic pigments [27].

Concerning the effect of the dietary inclusion of grape products on Haugh units, no consistent results seem to have been obtained among the few research studies available on this topic. On the one hand, Kara et al. [25] and Froes et al. [44] found that the Haugh units of the albumen remained unaffected despite the inclusion of GP in the diet of laying hens or quails, respectively, but on the other hand, Haugh units increased linearly with increasing dietary doses of grape seeds and grape seed extract in the work of Kaya et al. [30]. Therefore, further research is likely to be needed to confirm the effect produced on Haugh units by the dietary use of grape products.

### 4.3. Yolk Fatty Acid Profile

Yolk fatty acid profile was modified with the dietary inclusion of GP. Specifically, the inclusion of GP in the diet at 60 g/kg resulted in a decrease in the proportions of SFA and MUFA, while the proportion of PUFA was increased. Similarly, a dietary dose of GP at 20 g/kg increased the concentration of α-linolenic acid (a polyunsaturated fatty acid), while reducing that of oleic acid and elaidic acid (both MUFAs) in the egg yolks of quails [50]. Likewise, feeding laying hens with a diet containing 30 g/kg of grape seed meal enabled reductions in yolk concentrations of SFA, such as myristic acid and palmitic acid, and MUFA, such palmitoleic acid and oleic acid, while it increased the concentration of PUFA, such as linoleic acid and eicosatrienoic acid [31]. A slight reduction, although not statistically significant, in SFA proportion in the egg yolks of laying hens fed 20 g/kg of GP was reported along with a significant increase in the yolk concentrations of α-linolenic acid and docosahexaenoic acid (both PUFAs) in the study of Olteanu et al. [51]. All in all, these changes in the yolk fatty acid profile could convert conventional hen eggs into functional food, since the new fatty acid profile obtained could trigger positive health effects in human beings. Given that laying hens directly deposit into the egg yolk the dietary lipids they ingest [52], the changes in the fatty acid concentrations in the yolks of hens fed diets including grape products could be due to the different fatty acids provided by the oil contained in the grape seeds, as compared with other vegetable oil sources. Indeed, in the study of Çelik et al. [53], the egg yolk fatty acid composition was different depending on whether laying hens received flax seed oil or grape seed oil. With grape seed oil dietary inclusion, the yolk ω-6/ω-3 ratio increased, with this effect being dependant on the dose of grape seed oil included in the diet. The richness of grape seed oil in linoleic acid could have directly accounted for this effect. Nevertheless, the yolk concentrations of the different ω-6 and ω-3 fatty acids may also be influenced by the availability of the enzymes responsible for desaturation and elongation of fatty acids in the hen liver. There is actually a competition for desaturase enzymes between the metabolic pathways of ω-6 and ω-3 fatty acids [54]. For instance, the increased concentration of linolenic acid detected in the egg yolks of laying hens and quails fed GP at 20 g/kg limits the synthesis of arachidonic acid from linoleic acid, because linolenic acid competes with linoleic acid for the same Δ-6 desaturase enzymes.

### 4.4. Yolk Lipid Oxidation

In fresh eggs, no differences appeared among the various dietary treatments of the experiment for the MDA amount in the yolks. Values of MDA concentration obtained in the present study in the yolks of fresh eggs fell within the range of values found by other authors also in conventional fresh eggs of laying hens [16,25,47,55,56], but were around 50–75% lower than MDA concentrations measured in fresh eggs of hens fed ω-3 PUFA-enriched diets [16,57,58,59]. After four months of storage of eggs at room temperature, yolk MDA concentrations increased by ten times on average. Storage of eggs was carried out at room temperature instead of under refrigerated conditions and lasted four months, which may be deemed as a long period, in an attempt to deliberately cause lipid oxidation and perhaps enable the appearance of differences among the various dietary treatments of the study. Indeed, eggs do not become easily oxidised. As highlighted by Pike and Peng [60], egg yolk lipids underwent little oxidative deterioration even after 18 months of refrigerated storage. This is due to the presence of compounds with antioxidant activity (e.g., vitamin E, selenium, phospholipids) within the eggs. The MDA values measured in the four-month-stored eggs are in keeping with yolk MDA concentrations measured by other researchers after storage of hen eggs from two to six months [56,57,61]. In the stored eggs of the current research work, it was observed that the dietary inclusion of GP at 60 g/kg mitigated lipid oxidation during the storage, since stored eggs of this treatment showed an MDA concentration 30.5% lower than that of the control treatment. Kara et al. [25] also reported a reduction in egg yolk MDA concentration when laying hens were fed a diet containing 60 g/kg of GP. The average TBARS value of the other grape treatments of this work did not differ significantly from the TBARS value of the control group, which already constitutes a positive result taking into account that the control diet was supplemented with 100 mg/kg of vitamin E. It should be pointed out that, among the different grape product diets tested in this study, the GP 60 diet was the one presenting the highest concentration in grape extractable polyphenols. Resveratrol, a polyphenol with antioxidant effect present in red grapes, made possible a 28.6% reduction in the yolk MDA concentration in the eggs of quails fed diets supplemented with this phenolic compound [62]. Actually, in the latter study, egg yolk MDA concentration decreased linearly in response to increasing dietary resveratrol dose. As proved by the results of polyphenol digestibility of the present study, polymeric polyphenols from grapes can be hydrolysed in the intestine of birds. Actually, gut bacteria metabolise these polymeric polyphenols, giving rise to new phenolic compounds of smaller size that are easier to absorb and likely to reach animal tissues [63,64]. Once in the target tissues, these bioactive metabolites seem to exert an antioxidant effect by reducing radicals (α-tocopheroxyl) and thereby recycling the oxidised α-tocopherol [65].

## 5. Conclusions

Egg quality was improved with the intake of grape products, since the treatments including grape pomace, either at 30 or 60 g/kg, and grape extract at 1.0 g/kg increased both the yolk colour score and the albumen Haugh units. Dietary inclusion of grape pomace at 60 g/kg modified the fatty acid profile in the yolks by decreasing the proportions of saturated and monounsaturated fatty acids, while increasing that of polyunsaturated fatty acids. Additionally, in stored eggs of laying hens fed grape pomace at 60 g/kg, the yolks showed less oxidised lipids. Feeding grape pomace at 60 g/kg or grape extract at 0.5 g/kg improved the feed conversion ratio but decreased protein digestibility and reduced egg weight. In conclusion, the addition of grape products in the diet of laying hens improved the egg quality but reduced the egg weight. Dietary grape pomace showed antioxidant potential in egg yolk lipids higher than that of grape extract.

## Figures and Tables

**Table 1:** Proximate composition (g/100 g) of grape pomace.

Nutrients	Grape Pomace Composition
Humidity	8.2 ± 0.06
Crude protein	11.8 ± 0.08
Ether extract	7.5 ± 0.19
Crude fibre	14.6 ± 0.16
Neutral-detergent fibre	32.8 ± 1.78
Acid-detergent fibre	27.3 ± 1.47
Acid-detergent lignin	21.4 ± 1.87
Gross energy (kcal/kg)	4539 ± 9.9
Total extractable polyphenols (g gallic acid equivalents/100 g DM ^1^)	8.08 ± 0.06

Data are the mean of three determinations ± standard deviation. ^1^ DM = Dry matter.

**Table 2:** Ingredients and nutrient composition of experimental diets (g/kg as fed).

Ingredients	Experimental Diets				
	Control	GP ^1^ 30	GP 60	GE ^2^ 0.5	GE 1.0
Corn	547.5	515.4	483.6	547.5	547.5
Soybean	222.8	235.3	247.8	222.8	222.8
Sunflower meal	80.0	60.0	39.8	80.0	80.0
Sunflower oil	31.9	42.2	52.5	31.9	31.9
Grape pomace	−	30.0	60.0	−	−
Grape extract	−	−	−	0.50	1.0
Salt	3.4	3.4	3.3	3.4	3.4
Monocalcium phosphate	12.7	12.8	12.8	12.7	12.7
Calcium carbonate	85.7	85.2	84.8	85.7	85.7
Vitamin–mineral premix ^3^	5.0	5.0	5.0	5.0	5.0
L-Lysine	0.88	0.48	0.09	0.88	0.88
DL-Methionine	0.11	0.20	0.28	0.11	0.11
Vitamin E (mg/kg)	100	−	−	−	−
Celite ^4^	10.0	10.0	10.0	10.0	10.0
Analysed composition					
Crude protein	180	174	169	181	174
Crude fibre	66.5	70.2	71.3	65.7	66.1
Neutral-detergent fibre	123.0	124.2	128.8	120.7	118.5
Total extractable polyphenols (g GAE ^5^/kg)	2.50	4.91	7.42	2.65	2.86
Calculated composition					
Grape extractable polyphenols (g GAE/kg) ^6^	−	2.42	4.85	0.148	0.296
AME ^7^ (kcal/kg)	2750	2750	2750	2750	2750
Ether extract	57.5	68.8	80.1	57.5	57.5
Calcium	37.0	37.0	37.0	37.0	37.0
Available P	3.5	3.5	3.5	3.5	3.5
Sodium	1.5	1.5	1.5	1.5	1.5
Lysine	9.0	9.0	9.0	9.0	9.0
Meth + Cys	6.0	6.0	6.0	6.0	6.0

^1^ GP = Grape pomace. ^2^ GE = Grape extract. ^3^ Vitamin–mineral mix supplied the following per kilogram of diet: vitamin A, 12,320 IU; vitamin D_3_, 4620 IU; vitamin E, 15.4 IU; vitamin K, 3.08 mg; riboflavin, 6.16 mg; niacin, 46.2 mg; vitamin B_12_, 23.1 μg; pantothenic acid, 15.4 mg; folic acid, 0.31 mg; choline, 401 mg; Fe, as FeSO_4_, 50.4 mg; Zn, as ZnO, 71 mg; Mn, as MnO, 90 mg; Cu, as CuSO_4_, 7 mg; I, as ethylenediamine dihydroiodide, 0.7 mg; and Se, as Na_2_SeO_3_, 0.25 mg. ^4^ Celite Corp, Lompoc, CA. ^5^ GAE = gallic acid equivalents. ^6^ Calculated on the basis of the analyses of polyphenol concentration in GP and GE. ^7^ AME = apparent metabolizable energy.

**Table 3:** Effect of grape pomace (GP) and grape extract (GE) at different dietary concentrations (g/kg) on egg production, egg weight, egg mass, feed intake and feed conversion ratio in 50- to 54-week-old laying hens.

Diets	Daily Egg Production (%)	Average Egg Weight (g)	Daily Egg Mass (g/d)	Feed Intake (g/d)	Feed Conversion Ratio (g feed/g Egg Mass)
Control	82.3	68.5 ^a^	56.4	125.3 ^a^	2.18 ^a^
GP 30	82.6	67.4 ^b^	55.6	122.3 ^b^	2.15 ^ab^
GP 60	85.7	67.6 ^b^	57.8	120.5 ^bc^	2.07 ^bc^
GE 0.5	84.9	67.4 ^b^	56.9	118.8 ^c^	2.05 ^c^
GE 1.0	83.6	68.6 ^a^	57.2	121.9 ^b^	2.11 ^abc^
SEM ^1^	1.40	0.279	1.01	0.971	0.030
*p*-value ^2^	ns	**	ns	***	*

Different letters in the same column (a, b, c) indicate significant differences (*p* < 0.05). ^1^ SEM, standard error of the mean; each value represents the mean of three replicates per diet (25 hens per replicate). ^2^ ns: no significant effect (*p* > 0.05). * *p* < 0.05. ** *p* < 0.01. *** *p* < 0.001.

**Table 4:** Effect of grape pomace (GP) and grape extract (GE) at different dietary concentrations (g/kg) on the proportion of the different size classes of eggs in 50- to 54-week-old laying hens.

Diets	Proportion of XL ^1^ Eggs (%)	Proportion of L Eggs (%)	Proportion of M Eggs (%)	Proportion of S Eggs (%)	Proportion of Unmarketable 2 Eggs (%)
Control ^3^	21.1 ^ab^	72.2	6.49 ^c^	0.209	3.60
GP 30	14.8 ^c^	72.1	12.9 ^a^	0.198	3.96
GP 60	15.1 ^c^	72.5	12.2 ^ab^	0.243	2.90
GE 0.5	16.2 ^bc^	68.4	15.2 ^a^	0.242	2.37
GE 1.0	22.7 ^a^	68.6	8.49 ^bc^	0.210	3.21
*p*-value ^4^	**	ns	***	ns	ns

Different letters in the same column (a, b, c) indicate significant differences (*p* < 0.05). ^1^ The different size classes of eggs were: XL-very large, egg weight ≥ 73 g; L-large, 63 ≤ egg weight < 73 g; M-medium, 53 ≤ egg weight < 63 g; S-small, egg weight < 53 g. ^2^ Eggs were deemed not be suitable for sale if any of the following causes occurred: shell-less eggs, broken eggs, shell with deformities, eggs with abnormal shape or colour. ^3^ There were 3 replicates per diet, with 25 hens per replicate. ^4^ ns: no significant effect (*p* > 0.05). ** *p* < 0.01. *** *p* < 0.001.

**Table 5:** Effect of grape pomace (GP) and grape extract (GE) at different dietary concentrations (g/kg) on yolk colour score, Haugh units of the albumen and shell thickness in eggs of 50- to 54-week-old laying hens.

Diets	Yolk Colour Score	Haugh Units	Shell Thickness (μm)
Control	7.34 ^c^	76.4 ^b^	363.0
GP 30	7.98 ^b^	80.6 ^a^	368.0
GP 60	8.28 ^a^	80.2 ^a^	365.1
GE 0.5	8.06 ^ab^	77.0 ^b^	368.6
GE 1.0	8.18 ^ab^	81.6 ^a^	361.2
SEM ^1^	0.089	1.02	3.98
*p*-value ^2^	***	***	ns

Different letters in the same column (a, b, c) indicate significant differences (*p* < 0.05). ^1^ SEM, standard error of the mean; each value represents the mean of 120 eggs per diet (40 eggs per replicate). ^2^ ns: no significant effect (*p* > 0.05). *** *p* < 0.001.

**Table 6:** Effect of grape pomace (GP) and grape extract (GE) at different dietary concentrations (g/kg) on the yolk fatty acid profile in eggs of 50- to 54-week-old laying hens.

Diets	Saturated Fatty Acids (%)	Monounsaturated Fatty Acids (%)	Polyunsaturated Fatty Acids (%)	ω-6 Fatty Acids (%)	ω-3 Fatty Acids (%)	Ratio ω-6/ω-3
Control	32.9 ^ab^	41.4 ^ab^	25.7 ^bc^	24.8 ^bc^	0.682 ^a^	36.4 ^d^
GP 30	32.2 ^c^	41.6 ^ab^	26.2 ^b^	25.3 ^b^	0.627 ^c^	40.4 ^b^
GP 60	31.6 ^d^	39.5 ^c^	28.9 ^a^	28.0 ^a^	0.617 ^c^	45.4 ^a^
GE 0.5	32.6 ^bc^	40.7 ^b^	26.7 ^b^	25.8 ^b^	0.680 ^a^	37.9 ^c^
GE 1.0	33.0 ^a^	42.0 ^a^	25.0 ^c^	24.1 ^c^	0.657 ^b^	36.7 ^cd^
SEM ^1^	0.128	0.348	0.375	0.365	0.0075	0.457
*p*-value ^2^	***	***	***	***	***	***

Different letters in the same column (a, b, c, d) indicate significant differences (*p* < 0.05). ^1^ SEM, standard error of the mean; each value represents the mean of six samples per diet (two samples per replicate); each sample resulted from the pool of two yolks. ^2^ *** *p* < 0.001.

## Data Availability

The data presented in this study are available on request from the corresponding author.
